# Prospective evaluation of Ceftriaxone use in medical and emergency wards of Gondar university referral hospital, Ethiopia

**DOI:** 10.1002/prp2.383

**Published:** 2018-01-19

**Authors:** Asnakew Achaw Ayele, Begashaw Melaku Gebresillassie, Daniel Asfaw Erku, Eyob Alemayehu Gebreyohannes, Dessalegn Getnet Demssie, Amanual Getnet Mersha, Henok Getachew Tegegn

**Affiliations:** ^1^ Department of Clinical Pharmacy School of Pharmacy College of Medicine and Health Sciences University of Gondar Gondar Ethiopia; ^2^ College of Health Sciences Adigrat University Adigrat Ethiopia; ^3^ School of Medicine College of Medicine and Health Sciences University of Gondar Gondar Ethiopia

**Keywords:** anti‐microbial resistance, appropriateness of ceftriaxone, ceftriaxone, Ethiopia, Gondar University Referral Hospital

## Abstract

Ceftriaxone is among the most commonly utilized antibiotics owing to its high potency, wide spectrum of activity, and low risk of toxicity. It is used to treat different types of bacterial infections including pneumonia, bone infections, abdominal infections, Skin and soft tissue infections, urinary tract infections. However, evidence around the globe shows the misuse of Ceftriaxone**.** This study aimed at evaluating the appropriateness of ceftriaxone use in medical and emergency wards of Gondar university referral hospital (GURH), Northwest Ethiopia. A prospective, cross‐sectional study design was employed to evaluate the use of ceftriaxone. The medical records of patients who received ceftriaxone were reviewed prospectively between January 1 and March 30, 2017. Appropriateness of ceftriaxone use was evaluated as per the protocol developed from current treatment guidelines. A total of 390 patients’ medical records were reviewed. The utilization rate of ceftriaxone was found to be high with a point prevalence of 59%. Ceftriaxone was empirically used in 79.5% of cases. The most common indications of Ceftriaxone were respiratory tract infections (29.3%), central nervous system infections (24.1%), and prophylactic indications (16.4%). The mean duration of ceftriaxone therapy in our study was 11.47 days, with a range of 1‐52 days. More than two‐thirds (80.2%) of ceftriaxone use were found to be inappropriate and majority of unjustified ceftriaxone use emanated from inappropriate frequency of administration (78.3%), absence of culture and sensitivity test (68.7%), and duration of therapy (47%). Empiric treatment with ceftriaxone and the presence of coadministered drugs was significantly associated with its inappropriate use. The present study revealed a very high rate of inappropriate use of ceftriaxone which may potentially lead to emergence of drug‐resistant microorganisms and ultimately exposes the patient to treatment failure and increased cost of therapy.

**Abbreviations**
AP: aspiration pneumonia
CAP: community acquired pneumonia
FMHACA: Food, Medicine and Healthcare Administration and Control Authority of Ethiopia
GURH: Gondar university referral hospital
HAP: hospital acquired pneumonia
ICU: intensive care unit
SBP: spontaneous bacterial peritonitis
SPSS: Statistical Package for the Social Sciences
WHO: World Health Organization

## 1 INTRODUCTION

Antimicrobials play a paramount role in reducing the burden of infectious and communicable diseases all over the world. However, the curative power of these drugs is limited due to the development of resistance.^1^Emergence of antimicrobial resistance is a result of the use, over use and misuse of antibiotics.^2^ Antimicrobial‐resistant pathogens affect patient's outcome in different ways including a delay in the administration of appropriate antimicrobial therapy; and the antimicrobial therapies required to treat resistant pathogens can be toxic or inadequate.^3^Resistant infections also lead to an increase in of morbidity and mortality rate as well as prolong hospital stays. The problem is particularly severe in developing countries, where infectious diseases are more prevalent.^4^

Ceftriaxone is among the most commonly utilized antibiotics owing to its high potency, wide spectrum of activity, and low risk of toxicity. It is used to treat different types of bacterial infections including pneumonia, bone infections, abdominal infections, skin and soft tissue infections, and urinary tract infections. However, evidence around the globe shows the misuse of Ceftriaxone.^5^ A study conducted in Spain regarding the use of third generation cephalosporin, wherein ceftriaxone was the most frequently prescribed agent, found out that the cost of inappropriate antibiotic use was twice as much for patients who were treated appropriately.^6^Inadequate knowledge of treatment regimens and lack of diagnostic competence have contributed to incorrect drug choices, incorrect dose, adverse drug reactions, drug interactions, and use of more expensive drugs when less expensive drugs would be equally or more effective.^7^ In recognition to this problem, drug use evaluation has been recommended as a method for identifying inappropriate use that monitor, evaluate,and promote rational drug therapy.^8^ In Ethiopia, there are signs of irrational use of antibiotics by patients as well as by health care providers. According to the baseline survey conducted by Food, Medicine and Healthcare Administration and Control Authority of Ethiopia (FMHACA), about two‐thirds of patients (70%) patients who visited outpatient clinics have had one or more antibiotics prescribed with a percentage of irrational prescribing close to 40%.^2^Therefore, rational prescribing of antibiotics is vital as it reduces the emergence of antimicrobial resistance. Several studies were conducted in Ethiopia and elsewhere in the globe that evaluated the rational use of ceftriaxone injection.^6^, ^9^, ^10^, ^11^ However, previous studies were retrospective and use a smaller sample size. Taking the limitations of previous studies into consideration, a prospective cross‐sectional study design was employed and all wards of internal medicine and emergency wards were included in the present study. The aim of this study was, therefore, to evaluate the use and appropriateness of ceftriaxone in internal medicine and emergency wards of Gondar University referral hospital (GURH), Northwest Ethiopia.

## 2 MATERIALS AND METHODS

### 2.1 Study area

The study was conducted at the internal medicine and emergency wards of GURH, Northwest Ethiopia. GURH is a teaching hospital which acts as referral center for four district hospitals in the area. It has more than 1000 beds with a range of specialties, including internal medicine, pediatrics, surgery, gynecology, psychiatry, HIV care, and an outpatient clinic.

### 2.2 Study design

A prospective, cross‐sectional study design was employed to evaluate the use of ceftriaxone utilization in GURH. The medical records of patients who received ceftriaxone were reviewed prospectively between January 1 and March 30, 2017. The appropriateness of ceftriaxone utilization was evaluated, using a standard treatment protocol, which is developed after a thorough literature review regarding the rational use of ceftriaxone. Different literatures including the WHO and American Society of Health System Pharmacist's Criteria for Drug Use Evaluation 12, 13 as well as the Ethiopian Standard Treatment Guideline 14 were used. The content validity of the protocol was confirmed by a team of experts, including a senior physician with infectious disease expertise, a microbiologist, and a clinical pharmacist.

### 2.3 Population and sampling

The source populations were all patients admitted to medical and emergency wards of GURH and our study populations were all patients who were admitted in the medical and emergency wards of GURH between 1 and March 30, 2017. All adult (age ≥18 years) inpatients who took ceftriaxone at the medical and emergency wards of GURH between 1 and March 30, 2017 were included whereas, those with insufficient information and who did not give consent to participate were excluded. All the study participants included were followed until they stopped taking ceftriaxone. Single proportion formula was employed for determining the sample size by assuming a 95% confidence interval, *P* value of .5, and 10% contingency level. The sample size was adjusted based on the total number of patients who were estimated to take ceftriaxone in the 3‐month period based on retrospective hospital data (N = 1250). The final sample size was found to be 398 and every second patient encountered and who meets the inclusion criteria was included until the sample size was attained.

### 2.4 Data collection and management

Data were collected prospectively from medication charts by two of the principal investigators using a pretested data collection tool. The data collection tool included two parts. The first section includes information regarding age, sex, diagnosis, past medical history, abnormal laboratory, and diagnostic results. The second part includes information regarding ceftriaxone (indication, dose, frequency of administration and duration of therapy) as well as information regarding coprescribed medications. The accuracy and completeness of the collected data were verified continuously before the patient was discharged.

### 2.5 Statistical analysis

The final data collection tool was ensured for completeness, and responses were entered into and analyzed by the Statistical Package for the Social Sciences (SPSS) software version 21.0 for Windows. Frequency and percentage were used to express different variables. Univariate and multivariate logistic regression analysis were also employed to come up with factors associated with inappropriate use of ceftriaxone. Associations with significance level of less than 0.20 (*P *< .20) in the univariate analysis were included in the multivariate logistic regression analysis. Odds ratio with 95% CI were also computed along with corresponding *P*‐value (*P *< .05).

### 2.6 Ethical consideration

The study was approved by the ethical review committee of School of Pharmacy, University of Gondar. The data collected including name of the patient, the health care provider or drug products was kept anonymous.

## 3 RESULTS

### 3.1 Sociodemographic characteristics

Out of the 398 patients invited to participate, 390 gave consent and included in the study among which 50.5% were females. Most of the patients (94.1%) were adults in the age of 18‐65 with a mean age (with SD) of 34.5 ± 16.3. The sociodemographic profiles of study participants were depicted below (Table 1).

**Table 1:** Socio‐demographic profiles of study participants, GURH (*N*=390)

Variables	Category	Frequency (%)
Sex	Male	193 (49.5%)
	Female	197 (50.5%)
Age	18‐65	367 (94.1%)
	≥65	23 (5.9%)
Department	Internal medicine	316 (81%)
	Emergency	74 (19%)
Unit of admission	Non‐ICU^a^	352 (90.2%)
	ICU	38 (9.8%)
Length of hospital stay	0‐7 days	117 (30%)
	8‐14 days	157 (40.2%)
	>14 days	116 (29.7%)

a ICU, intensive care unit.

### 3.2 Ceftriaxone use and appropriateness of therapy

The utilization rate of ceftriaxone was found to be high with a point prevalence of 59%. In 63.8% of cases, ceftriaxone was indicated as a first‐line therapy. However, in most cases (79.5%), ceftriaxone was prescribed empirically. The most common indications of Ceftriaxone were respiratory tract infections (29.3%), central nervous system infections (24.1%) and prophylactic indications (16.4%). Regarding the dosage and duration of ceftriaxone use, 1 g dose was used frequently (70%) and twice‐daily dosing (76.9%) was the most frequently used for administration (Table 2 and 3).The mean duration of ceftriaxone therapy in our study was 11.47 days, with a range of 1‐52 days. In most of the cases (85.9%), culture and sensitivity test was not performed due to many reasons, including earlier initiation of therapeutic regimen (22.1%) and prophylactic use of ceftriaxone (19.2%).However, among 55 cases in which the tests were performed, the growth of resistant organisms was observed in 39 (71%) of the cases.

**Table 2:** Prescription pattern of ceftriaxone in the study participants, GURH, 2017 (N=390)

Characteristics	Category	Frequency (%)
Indication of ceftriaxone	Primary	249 (63.8%)
	Alternative	126 (32.4)
	Not indicated	15 (3.8%)
Type of treatment	Therapeutic, Empiric	310 (79.5%)
	Therapeutic, Specific	16 (4.1%)
	Prophylactic	64 (16.4%)
Reasons for ceftriaxone use	Respiratory tract infection	114 (29.3%)
	Prophylactic indications	64 (16.4%)
	Skin, soft tissue and bone infection	16 (4.1%)
	Central nervous system infection	94 (24.1%)
	Sepsis and septic shock	6 (1.5%)
	Cardiovascular infection	17 (4.3%)
	Urinary tract infection	33 (8.5%)
	Gastro‐intestinal infection	34 (8.7%)
	No indication	12 (3.1%)

**Table 3:** Dosing and duration of treatment with ceftriaxone in the study participants, GURH, 2017 (N=390)

Variable	Category	Frequency (%)
Dose (gm)	1	273 (70%)
	1.5	16 (4.1%)
	2	101 (25.9%)
Daily dose (gm)	1	6 (1.5%)
	2	300 (76.9%)
	3	6 (1.5%)
	4	78 (20%)
Duration (days)	1	20 (5.1%)
	2‐7	101 (25.9%)
	8‐14	145 (37.2%)
	15‐21	78 (20%)
	>21	46 (11.8%)

**Figure 1:**
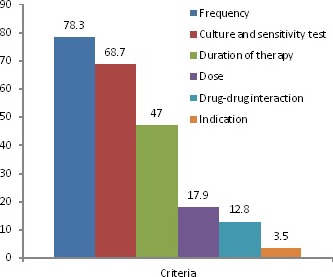
Criteria referenced inappropriate use of ceftriaxone, GURH, 2017 (N = 390)

More than two‐thirds 313 (80.2%) of ceftriaxone use were reported to be inappropriate and majority of unjustified ceftriaxone use emanated from inappropriate frequency of administration (78.3%), absence of culture and sensitivity test (68.7%), and duration of therapy (47%)(Figure 1).

Inappropriate use of ceftriaxone was considerably higher in the emergency ward than medical wards (93.2% and 72.2%, respectively) and a higher proportion of inappropriate use was recorded in the treatment of pneumonia and spontaneous bacterial peritonitis (SBP) (Table 4).

**Table 4:** Appropriateness of ceftriaxone use among the most common indications, GURH, 2017 (N = 390)

Indication	Appropriate N (%)	Inappropriate N (%)
Pneumonia, CAP	2 (5%)	28 (95%)
Pneumonia, AP	3 (9.1%)	30 (89.9%)
Pneumonia, HAP	0	35 (100%)
Pyogenic meningitis	35 (43.7%)	45 (56.3%)
Brain abscess	6 (42.8%)	8 (57.2%)
Sepsis	4 (66.7%)	2 (33.3%)
Cellulitis	4 (36.3%)	7 (63.7%)
SBP	6 (23.1%)	20 (76.9%)

AP, aspiration pneumonia; CAP, community acquired pneumonia; HAP, hospital acquired pneumonia; SBP, spontaneous bacterial peritonitis.

### 3.3 Coadministered drugs

**Figure 2:**
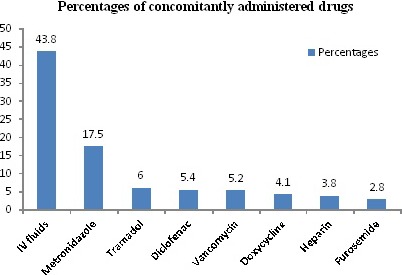
Drugs concomitantly prescribed with ceftriaxone, GURH, 2017 (N = 390)

The most frequently coadministered drugs with ceftriaxone were IV fluids (43.8%), metronidazole (17.5%), tramadol (6%), and Diclofenac (5.4%). Among the coadministered IV fluids, 69 (40.3%) were ringer lactate which could potentially interact with ceftriaxone as it contains calcium (Figure 2).

### 3.4 Factors associated with inappropriate ceftriaxone use

Using bivariate logistic regression, factors that were associated with inappropriate use of ceftriaxone in the study population included co morbidity, days of hospital stay, and the type of therapy with ceftriaxone and presence of co administered drugs. Having other variables controlled the type of therapy with ceftriaxone and presence of coadministered drugs remained to be significant in the multivariate logistic model. Accordingly, Empiric treatment with ceftriaxone (AOR = 22.57; 95% CI, [4.66‐41.47]), and the presence of coadministered drugs (AOR = 4.12; 95% CI, [1.62‐8.05]) was significantly associated with its inappropriate use (Table 5).

**Table 5:** Factors associated with inappropriate use of ceftriaxone using multivariate logistic regression, GURH, 2017 (N = 390)

Variable	Appropriateness			
	No (%)	Yes (%)	AOR (95% CI)	*P* value
Gender				
Male	175 (90.7%)	18 (9.3%)	0.87 (0.49–1.69)	.631
Female	138 (70%)	59 (30%)	1.00	
Age				
18‐65	301 (82%)	66 (18%)	‐	.300
>65	12 (52.2%)	11 (47.8%)	‐	
Department				
Emergency	69 (93.2%)	5 (6.8%)	0.71 (0.32:1.69)	.132
Internal medicine	244 (77.2%)	72 (22.8%)	1.00	
Unit				
Non‐ICU	285 (81%)	67 (19%)	0.91 (0.34–1.92)	.347
ICU	28 (73.7%)	10 (22.3%)	1.00	
Treatment type				
Empiric	276 (89%)	34 (11%)	22.57 (4.66‐41.47)^a^	.001^a^
Specific	4 (25%)	12 (75%)	1.00	
Days of hospital stays				
0–7 days	102 (87.2%)	15 (12.8%)	‐	.505
8–14 days	120 (76.4%)	37 (23.6%)	‐	
>14 days	91 (78.4%)	25 (21.6%)	‐	
Coprescribed drugs				
0	5 (41.7%)	7 (58.3%)	1.00	.002^a^
≥1	308 (81.5%)	70 (18.5%)	4.12 (1.62–8.05)^a^	

a statically significant.

## 4 DISCUSSION

This study revealed a prevalence rate of 59% ceftriaxone use. The finding in our study is comparable with the studies done in Addis Ababa (58%) and Spain (66%).^6^, ^15^However, the study done in India reported a relatively high prevalence (72%).^16^In contrast, a lower utilization rate of ceftriaxone was reported in Tehran (34%).^17^A higher prevalence of ceftriaxone use could be partially explained by the fact that ceftriaxone has been recognized as a drug of choice owing to its excellent bioavailability, effectiveness, and low toxicity profile.^18^, ^19^The variation in the degree of ceftriaxone use among different countries might be owing to differences in the availability of this drug and use of other cephalosporin antibiotics. Ceftriaxone was used as an empiric treatment in 79.5% of cases, which is comparable to the study done in Addis Ababa (87.3%),^19^ but higher compared to the study conducted in the West Indies (67.9%).^20^However, the latter study takes into account other antibiotics in addition to ceftriaxone for calculating the rate of empiric antibiotic use. The most common indications of Ceftriaxone were respiratory tract infections (29.3%) followed by central nervous system infections (24.1%) and prophylactic indications (16.4%). Our finding corroborates with the study done in Dessie,^11^ where respiratory tract infections, principally pneumonia (36.4%), were the commonest indication for ceftriaxone.

In our study, culture and sensitivity test was not performed in most of the cases (85.9%) and among 55 cases in which the tests were performed, the growth of resistant organisms was observed in 71% of the cases. The rate of performing culture and drug sensitivity test was very low compared to the study conducted in Addis Ababa^15^ and Korea.^5^ Some of the possible reasons are the poor quality of the microbiology laboratory of GURH and the high cost of culture and drug sensitivity, which is usually not affordable by the patient. Besides, it will take an average of 4 days for the laboratory results to be available, which could compromise the health status of the patient. Due to this, most of the physicians prefer treating the patient empirically than sending the culture and drug sensitivity test to laboratory. However, 39 out of 55 cases (71%) in which drug sensitivity test was performed develop resistance to ceftriaxone. This finding is high compared to studies done in other regions of Ethiopia and elsewhere in the globe,^15^, ^21^, ^22^ even though the number of cases which are sent for sensitivity test and the rate of ceftriaxone use in those studies is different from our study.

It is interesting to note that twice‐daily administration accounted for 76.9% of the cases and at the same time, frequency of administration was found to be the leading cause of inappropriate ceftriaxone use which is observed in 78.3% of the cases. This finding was similar in the study done in Addis Ababa^15^ and USA.^23^The mean duration of ceftriaxone therapy in our study was 11.47 days, with a range of 1‐52. Duration of therapy with ceftriaxone was the third cause of inappropriate use (47%) only next to frequency of therapy and culture and sensitivity test. The result is comparable to studies done in Addis Ababa and Korea,^5^, ^15^ but relatively lower values was also reported in other studies.^10^, ^11^ The differences in duration of ceftriaxone therapy could be partially explained by the fact that most patients visiting GURH are referred from other neighboring hospitals and are terminally ill, resulting in physicians going for long duration therapy. Noncompliance to the current recommended treatment guideline was also observed as 59% of patients continue taking ceftriaxone injection, while switching to oral medication was appropriate for these patients. In addition, ceftriaxone was used for more than 4‐8 days for prophylactic purpose, while evidence and international literature recommended a stat (once) dosing of ceftriaxone is enough for prophylaxis.^24^, ^25^Among the coprescribed drugs, IV fluids (43.8%) took the first place followed by Metronidazole (17.5%), and tramadol (6%). From the coprescribed drugs, Ringer lactate was prescribed in 69 of the cases, which could potentially interact with ceftriaxone as it contains calcium. Similarly, heparin was also prescribed which is thought to interact moderately with ceftriaxone, thereby predisposing patients for bleeding.

More than two‐thirds (80.2%) of ceftriaxone use were reported to be inappropriate. The result is comparable with the study done in Addis Ababa (87.9%) 15 and Tehran (85.3%),^17^ but higher than the study done in Mekelle (64.2%)^10^ and Dessie (46.2%).^11^ The difference in rate of inappropriate use of ceftriaxone among different studies is largely attributed to the criteria used to evaluate the appropriateness of ceftriaxone use as most of the local retrospective studies used only the Ethiopian standard treatment guideline. In the multivariate logistic model, empiric treatment with ceftriaxone was significantly associated with its inappropriate use. The presence of coadministered drugs also significantly affected the appropriate use of ceftriaxone. However, other variables like age, sex, comorbidity, age, and units of admission were not associated with inappropriate use of ceftriaxone unlike some other studies which reported a significant association between inappropriate use of ceftriaxone and sex.^26^ This may be due to the inclusion of more female patients in the latter study.

### 4.1 Limitations

The study has some limitations that should be considered while interpreting the results. The present study excludes departments other than emergency and internal medicine such as surgical ward, which could have underestimated the overall findings. Since there are limited local prospective studies regarding ceftriaxone use, most of the comparisons were made with retrospective studies. Finally, the present study did not consider renal function of patients which is important to interpret dose and frequency.

## 5 CONCLUSION

The present study revealed a very high rate of inappropriate use of ceftriaxone which may potentially lead to the emergence of drug‐resistant microorganisms and ultimately exposes the patient to treatment failure and higher cost of therapy. Empiric treatment with ceftriaxone and the presence of coadministered drugs was significantly associated with its inappropriate use.

Generally, prescribers should adhere to current evidence‐based guidelines and reserve ceftriaxone only for proven or strongly suspected infections. Periodic and continuous evaluation of antibiotics use is also warranted as it will reduce the injudicious use of antibiotics including ceftriaxone. Establishing antimicrobial stewardship program in the hospital is also recommended for sustainable and rational use of antibiotics.

## DISCLOSURE

All the authors declare that they have no competing interest.
